# Decoding promoter activity from DNA sequence using pre-trained language models

**DOI:** 10.1038/s41598-026-61483-w

**Published:** 2026-07-14

**Authors:** Christophe Jung

**Affiliations:** Gene Center Munich, Department of Biochemistry, Quantitative and Molecular Biology (QMB), Ludwig-Maximilians-Universität München, Feodor-Lynen- Strasse 25, 81377 München, Germany

**Keywords:** Biotechnology, Computational biology and bioinformatics, Genetics, Molecular biology

## Abstract

Promoter architecture plays a central role in transcriptional regulation, but predicting promoter activity directly from DNA sequence remains challenging. Here, we tested whether transformer-based DNA language models can learn regulatory logic encoded in *Drosophila* core promoters. We fine-tuned the pretrained DNA language model DNABERT-2 using a synthetic core promoter dataset measured in S2 cells with luciferase reporter assays. The model predicted promoter activity well when biological replicates were split between training and test data (R² ≈ 0.91), and retained meaningful performance when test promoter sequences were fully excluded from training (R² ≈ 0.64). Model interpretation using SHapley Additive exPlanations (SHAP)^1^ showed that predictive sequence features matched known promoter elements, including INR, TATA box, DRE, Ohler and MTE/DPE motifs, with position-dependent effects consistent with promoter architecture. Incorporating hormonal activation and nucleosomal context enabled sequence and biological context to be modeled in a unified framework. Gene-wise cross-validation showed promoter-specific generalization across most promoters with promoter-specific differences in accuracy. Applied without retraining to independent *Drosophila* embryo promoter data, the model captured partial in vivo activity trends. These results show that DNA language models can learn interpretable promoter sequence rules from controlled datasets, while accurate in vivo prediction will require broader regulatory context.

**Supplementary Information:**

The online version contains supplementary material available at 10.1038/s41598-026-61483-w.

## Introduction

Accurate control of gene expression is fundamental to development and cellular function^1,2^. In metazoans, transcription initiation is largely determined by the core promoter, a region of approximately 150 bp surrounding the transcription start site (TSS) that recruits RNA polymerase II and the general transcription machinery^3,4^. Core promoters integrate several types of regulatory information, including short sequence motifs^5,6^, motif strength and spacing^7–9^, DNA shape^10^,and local chromatin context^11–13^. In *Drosophila melanogaster*, systematic experimental work has identified a diverse set of core promoter elements (CPEs), such as the Initiator (INR)^5^, TATA-box^14^, downstream promoter element (MTE/DPE)^13^, Ohler motifs^7,15^,and others, which combine in distinct architectures associated with developmental or constitutive gene expression^13^.

Massively parallel reporter assays (MPRAs)^16,17^have enabled direct and quantitative testing of promoter sequences at large scale. These studies showed that promoter activity can often be explained by the additive effects of individual motifs, with additional influence by motif position^17^, sequence context^17^, nucleosome positioning^15^,and hormonal signaling^13^.

Despite these advances, building predictive models that generalize across promoters and genes remains difficult. In our previous work^13^, promoter activity in *Drosophila*S2 cells was successfully modelled using linear regression with motif-based features, demonstrating that expression can be largely explained by additive contributions of individual motifs. However, such models depend on predefined motif annotations and do not test whether promoter regulation can be learned directly from DNA sequence. Recent non-language-model approaches, including PARM^18^, Puffin^19^, and PromoterAI^20^, have also shown that deep learning can predict promoter activity or promoter-variant effects from sequence using alternative architectures and training objectives. These studies provide an important benchmark for sequence-to-function modelling, while leaving open how pretrained DNA language models perform on small, controlled promoter libraries and whether their predictions can be interpreted at the level of core promoter features.

Transformer-based DNA language models offer a powerful alternative for modelling regulatory DNA^21,22^. DNABERT-2^22^ adapts the BERT (Bidirectional Encoder Representations from Transformers)^23^ architecture to genomic sequences by learning contextualized representations of overlapping strings of size k, called k-mers, through masked-language pretraining. This allows the model to capture short sequence motifs as well as their positional and combinatorial relationships without motif predefinitions.

Sequence-based language models allow us to test whether core promoter activity can be predicted using only the DNA sequence. They may also help explain the biological features that influence promoter activity. These models have been useful for tasks such as predicting chromatin accessibility and enhancers^24^. However, their ability to accurately predict promoter activity from small synthetic promoter libraries still needs careful testing. It is also important to assess whether they can provide meaningful biological insights at the promoter level.

**Fig. 1:**
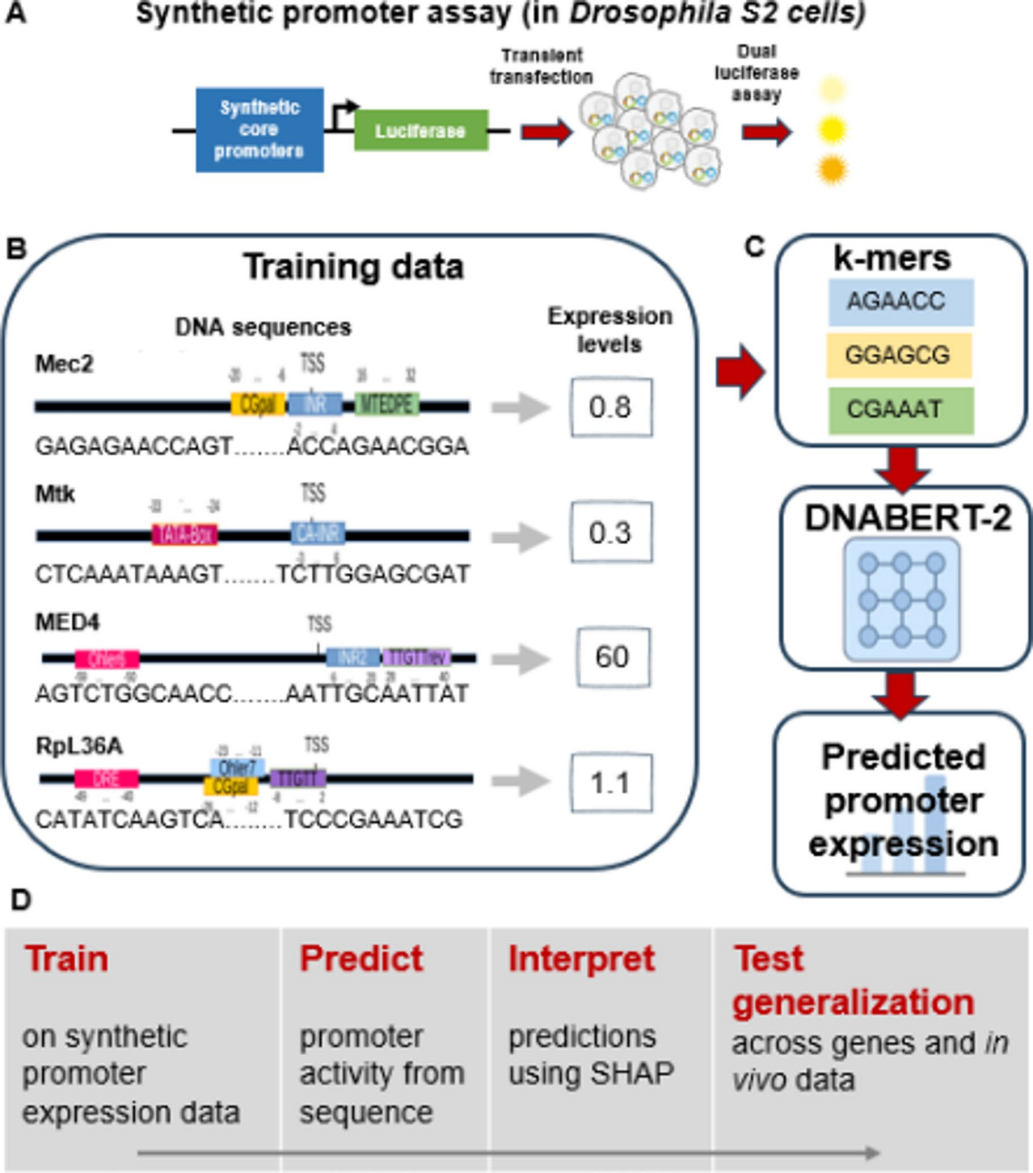
Experimental system and modeling overview. **(A)** Schematic of the dual-luciferase reporter assay used to quantify synthetic core promoter activity in *Drosophila* S2 cells (data from Qi et al.^13^). **(B)** Training dataset consisting of designed promoter DNA sequences paired with experimentally measured expression levels, used for model fine-tuning. **(C)** Schematic depicting the k-merisation process of the input DNA sequence, that will be used by the DNABERT-2 algorithm for promoter expression predictions. **(D)** Computational workflow: promoter sequences are used to train a sequence-based model, predictions are interpreted using SHAP, biological context is integrated, and model generalization is assessed using gene-wise validation and independent in vivo data.

To assess whether core promoter activity can be predicted directly from DNA sequence, we used a large synthetic promoter dataset that we previously generated and characterized in *Drosophila*S2 cells^13^. This dataset consists of systematically designed core promoter variants measured with a quantitative dual-luciferase reporter assay, providing precise and reproducible expression values under controlled conditions, both with and without ecdysone (Ecd) hormonal stimulation (Fig. 1A, B). The scale and experimental control of this dataset make it well suited for training and evaluating sequence-based models.

We used these sequence–expression pairs to fine-tune DNABERT-2 to predict promoter activity directly from DNA sequence (Fig. 1C). We evaluated model performance on held-out data and confirmed robustness using control experiments. To understand what the model learned, we applied SHAP^25^ to measure how individual sequence features contributed to predicted expression levels. This allowed us to identify the sequence elements that drive promoter activity in the model.

We further examined how promoter-intrinsic sequence logic interacts with biological context by incorporating Ecd activation and flanking − 1/+1 nucleosomal sequences into the analysis. Finally, we tested model generalization using gene-wise cross-validation and independent in vivo promoter activity data from Drosophila embryos (Fig. 1D).

Together, our results show that transformer-based DNA language models can predict core promoter activity from sequence in controlled settings and recover sequence features consistent with known promoter architecture. The results support the use of these models for prediction and hypothesis generation, but they do not constitute a complete mechanistic explanation of promoter regulation.

## Results

### Fine-tuning DNABERT-2 on synthetic drosophila promoter libraries

We fine-tuned DNABERT-2 on a subset of our previously generated synthetic promoter dataset^13^, focusing first on core promoter variants measured in a fixed nucleosomal context (referred to as N1.x and N7.y, corresponding to − 1 and + 1 nucleosomal sequences^13^, respectively), and without Ecd induction. The dataset comprised approximately 700 unique promoter sequences (131 bp core region) with approximately 2,600 quantitative luciferase measurements, averaging approximately 2.8 biological replicates per sequence. Expression values were log2-transformed before modelling. For replicate-level training, replicate measurements were kept as separate data points. For analyses requiring a single value per promoter sequence, we used the median expression value for each promoter sequence.

DNABERT-2 supports native subword-based tokenization schemes that generate many short and length-variable sequence tokens^22^. While these representations yielded comparable predictive performance in preliminary experiments, they strongly reduced interpretability because sequence features were split across many tokens. In contrast, fixed 6-mer tokenization provided a simpler and more biologically intuitive representation, aligned with known core promoter motifs and enabled robust interpretation using SHAP. We therefore adopted 6-mer tokenization for all analyses (Methods).

We first randomly split individual promoter activity measurements into 90% for training and 10% as a held-out test set for final model evaluation. This replicate-level split preserves biological replicate variation and estimates performance within the same assay setting, but it can place replicate measurements of the same promoter sequence in both training and test sets. We therefore added a stricter sequence-disjoint split in which all measurements from the same promoter sequence were assigned together to either training or test data. This prevents exact promoter-sequence overlap between the two sets and provides a more conservative test of generalization to unseen promoter sequences.

**Fig. 2:**
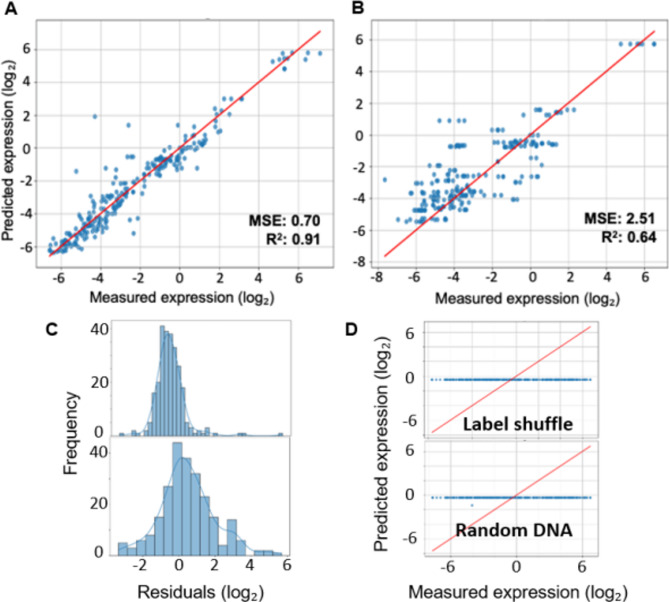
Accurate prediction of promoter activity from DNA sequence. (**A**) Scatter plot comparing predicted and measured promoter activity (log₂ scale) for promoter activity measurements randomly held out from training using the replicate-level train/test split. The red diagonal indicates perfect prediction. (**B**) Sequence-disjoint evaluation as a stricter split in which all replicate measurements from the same promoter sequence were assigned together to either training or test data. (**C**) Residuals from the replicate-level model (upper panel) and the sequence-disjoint evaluation (lower panel) are shown to assess bias across the activity range. (**D**) Negative controls. Predicted versus measured activity after fine-tuning on randomly shuffled expression labels (upper panel), or after replacing promoter sequences with random DNA of matched length and composition (lower panel). In both cases, predictive performance collapses toward baseline, consistent with loss of the true sequence-expression relationship.

Under the original replicate-level split, the sequence-only model achieved strong predictive performance on the test set after fine-tuning (R² ≈ 0.91; MSE ≈ 0.70), indicating close agreement between predicted and measured promoter activity across a wide dynamic range (Fig. 2A and C upper panel, and Supplementary Fig. 1 A). Under the sequence-disjoint split, performance decreased as expected but retained clear predictive signal (R² ≈ 0.64; MSE ≈ 2.51; Fig. 2B and C lower panel, and Supplementary Fig. 1B). This shows that the replicate-level split mainly measures how well the model fits the assay data, while the sequence-disjoint split gives a stricter test of how well the model predicts new promoter sequences.

To confirm that the model learned sequence-expression relationships, we performed negative control experiments. In the label-shuffle control, the expression labels were randomly permuted across promoter sequences and the model was fine-tuned again on this shuffled dataset. Because the link between sequence and expression was removed, the model could not learn meaningful differences between sequences. It therefore predicted values near the average expression level, so the predictions collapsed into a narrow range (Fig. 2D upper panel, and Supplementary Fig. 1 C). When promoter sequences were replaced with random DNA of matched length and composition, predictive performance also decreased to baseline levels (Fig. 2D lower panel, and Supplementary Fig. 1D). These controls indicate that DNABERT-2 captures real sequence-expression relationships rather than relying only on memorization or non-specific sequence patterns.

We also compared DNABERT-2 with two simpler sequence-based models under the same sequence-disjoint split. A 6-mer ridge regression model, in which each promoter was represented by its 6-mer content^26^, achieved R² ≈ 0.50 and MSE ≈ 3.46. A simple Convolutional Neural Network (CNN) trained on one-hot encoded promoter sequences^27^ achieved R² ≈ 0.61 and MSE ≈ 2.70 (Methods). Under the same split, DNABERT-2 achieved R² ≈ 0.64 and MSE ≈ 2.51. Thus, much of the predictive signal can be captured by local sequence features, and DNABERT-2 does not clearly outperform a simple CNN in this conservative evaluation. However, DNABERT-2 provides 6-mer-level attributions (see below) that are easier to relate to known core promoter motifs than the base-level filters learned by the CNN.

### Model interpretability reveals promoter regulatory logic

Transformer-based models learn attention weights that quantify how strongly individual sequence tokens attend to one another when making predictions^21,22^. In principle, attention maps can highlight regions of a DNA sequence that the model considers informative. However, for promoter activity prediction, attention maps alone are difficult to interpret biologically. DNABERT-2 contains many layers and attention heads, yielding complex patterns that cannot be easily linked to specific regulatory elements (Supplementary Fig. 2).

Therefore, we used SHAP to obtain direct, quantitative insight into the sequence features driving the model predictions^25^. SHAP is a widely used standard explainability method that assigns an additive contribution value to each input token according to its effect on the predicted output. We chose SHAP instead of the commonly used LIME method^28^ because SHAP provides additive scores for individual 6-mers. These scores can be compared across promoters and directly linked to motif-like sequence tokens. In contrast, LIME explains predictions by introducing local perturbations and fitting a simpler model that approximates the behavior of the original model around that specific input. This approach may be harder to interpret for overlapping DNA 6-mers, because perturbing one token, that is, one 6-mer, can also affect the surrounding sequence context. SHAP values should therefore be interpreted as model-based feature attributions, not as evidence that a sequence element directly causes promoter activity.

**Fig. 3:**
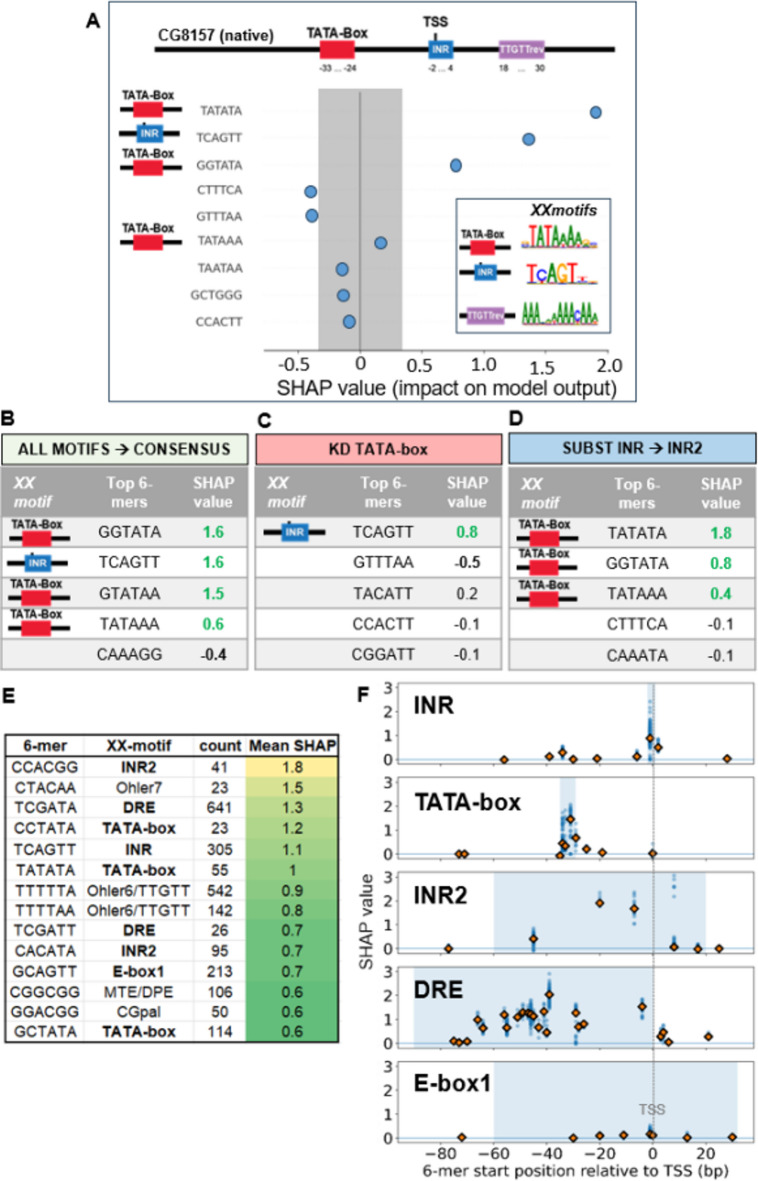
SHAP analysis reveals promoter regulatory logic learned by the model. **(A)** SHAP attribution scores mapped along the sequence of a representative promoter (native CG8157). SHAP-enriched 6-mers were assigned to motif classes (on the left hand-side) based on sequence similarity to *XXmotif* consensus motifs. The upper panel indicate the promoter architecture with motif positions as found by *XXmotif*^13^. The inset shows the binding preferences for the three motifs identified by *XXmotif* in CG8157. **(B-D)** SHAP analysis of different promoter variants with experimentally introduced motif changes and measured activity. Shown are representative cases for CG8157 including **B** replacement of all motifs by their consensus sequences, **C** motif knockout of the TATA-box, and **D** motif substitution (Initiator replaced by INR2). Compared to the native promoter profile shown in **A**, replacement by consensus motifs results in stronger and more focused SHAP signals, disruption of the TATA-box leads to a loss of SHAP signal at the corresponding positions, while functional substitution redistributes SHAP contributions and INR2 does not recover the loss of INR. **(E)** Table of the most informative 6-mers across the dataset, ranked by mean SHAP contribution. For each 6-mer, the associated *XXmotif* annotation, occurrence count, and mean SHAP value are shown. Highly ranked 6-mers correspond to canonical core promoter elements. Motifs analyzed in **F** are highligthed in bold. (**F**) SHAP attribution profile across the promoter sequence. Positive SHAP values indicate sequence features that increase the model-predicted promoter activity. Blue shaded regions mark motif genomic regions previously identified by the bioinformatic analysis of Qi et al.^13 ^The co-localization of high SHAP contributions with strong annotated motif regions indicates that the model captures sequence features corresponding to functionally relevant promoter motifs^. ^ In contrast, E-box 1 showed only a weak contribution to promoter activity in our experimentally measured promoter variants^13^, indicating that this annotated motif has limited functional strength in our assay.

To quantify how each input affects the prediction, we applied SHAP to the fine-tuned model (Methods), assigning importance scores to each 6-mer based on its contribution to predicted expression (Fig. 3). Across the dataset, SHAP consistently highlighted short sequence elements matching known core promoter motifs. SHAP-enriched 6-mers were associated with known core promoter motifs by comparison to *XXmotif*consensus sequences^7,13^, allowing for limited sequence variation (Methods).

For the developmental promoter CG8157, SHAP-enriched regions showed strong agreement with experimentally defined promoter elements identified by *de novo* motif discovery (Fig. 3A). INR and TATA box motifs contributed strong positive effects, whereas the TTGTTrev motif did not appear among the top SHAP values, consistent with its previously reported weakly repressive role.

Analysis of experimentally generated promoter variants showed that SHAP attributions changed predictably with motif identity and motif strength (Fig. 3B-D). These variants were distinct from the promoter shown in Fig. 3A and contained defined motif perturbations with experimentally measured activity. Replacing native motifs with stronger consensus motifs increased the positive SHAP signal at the motif positions (Fig. 3B). TATA-box knockdown strongly reduced the TATA-associated SHAP signal and lowered positive contributions across the promoter (Fig. 3C). Substitution of the Initiator motif with the INR2 variant redistributed the SHAP signal to the altered Initiator context while preserving partial predicted activity (Fig. 3D). These findings indicate that the model assigns activity to biologically meaningful motif changes in experimentally perturbed promoters. Nevertheless, SHAP values remain model-based feature attributions, and causal interpretation should be grounded in the experimental activity measurements and controlled comparisons between promoter variants.

A global analysis of the most informative 6-mers further supported the conclusion that high-impact tokens corresponded to well-known core promoter motifs, including INR, INR2, TATA box, DRE, Ohler6/7, and MTE/DPE (Fig. 3E). Differences in SHAP value distributions across motif classes reflected known positional constraints and regulatory roles (Supplementary Fig. 1 F).

We next examined whether SHAP attributions aligned with motif genomic regions previously identified by the bioinformatic analysis of Qi et al.^13^ In Fig. 3F, blue shaded regions indicate these annotated motif regions. High SHAP values were enriched at expected motif positions: INR-associated 6-mers were concentrated near the TSS, TATA-box-associated 6-mers were enriched upstream of the TSS, and INR2 / DRE-associated 6-mers showed broader positional behavior consistent with weaker positional constraint. Importantly, our experimentally measured promoter variants showed that E-box 1 has only a weak effect on promoter activity, indicating that this annotated motif has limited functional strength in our assay^13^. Consistent with this, the SHAP signal at E-box 1 was weaker than at stronger motif regions. Thus, the attribution profile reflects not only the presence of annotated motif regions, but also their experimentally observed contribution to promoter activity. These results indicate that the model captures not only motif-like sequences, but also differences in their position-dependent contributions to promoter activity. However, SHAP results should be viewed as model-based hints, not as proof of a regulatory mechanism.

### Integrating biological context modulates prediction accuracy

**Fig. 4:**
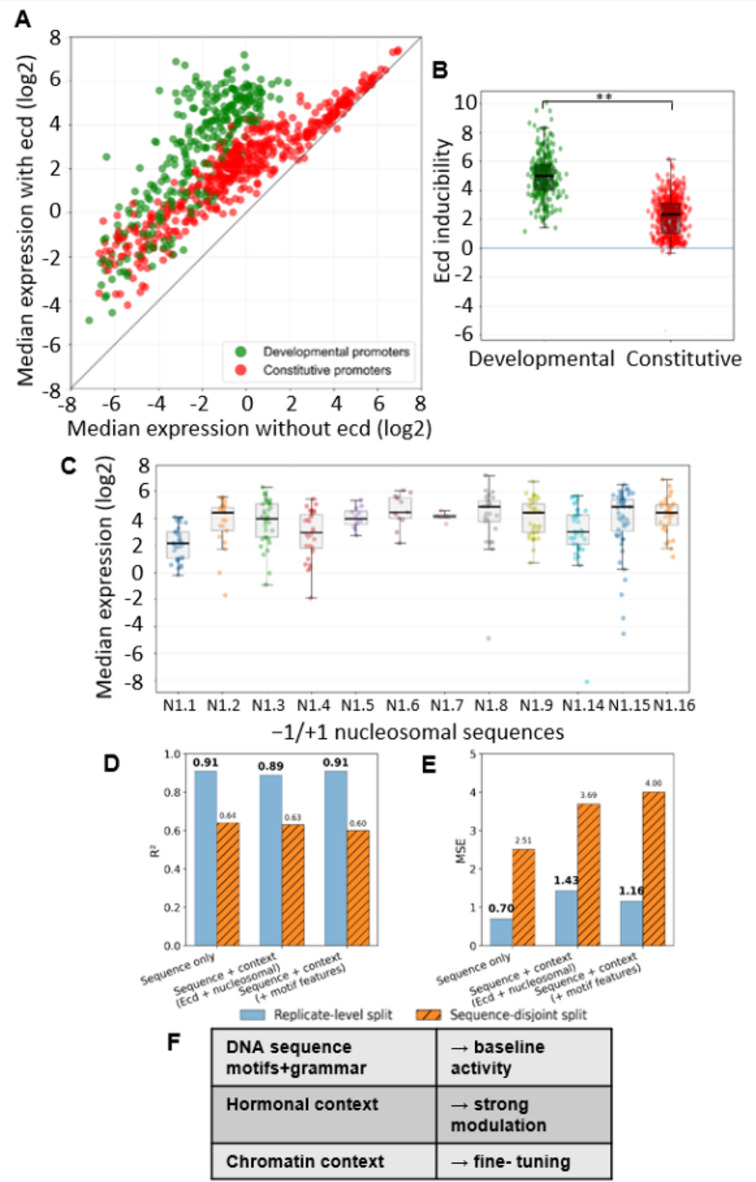
Biological context modulates promoter activity and prediction accuracy. **(A)** Hormonal induction of promoter activity. Median promoter activity measured in the presence of Ecd (log₂ scale) plotted against median activity measured without Ecd (log₂ scale) for all our synthetic promoter variants. Each point represents one promoter. The diagonal indicates equal expression in both conditions. Data are derived from Qi et al.^13^. **(B) **Ecdysone inducibility (Δlog₂ expression) grouped by promoter class. As previously reported^13^, developmental promoters show significantly higher inducibility than constitutive promoters (*p* = 0.0054). Statistical significance was assessed using a two-sided Wilcoxon rank-sum test. * *p* < 0.05; ** *p* < 0.01; *** *p* < 0.001; ns, not significant. Boxes indicate median and interquartile range; points represent individual promoters. **(C)** Effect of − 1/+1 nucleosomal sequences on promoter activity. Median promoter activity (log₂ scale) plotted for different − 1/+1 nucleosomal sequences flanking the core promoter (N1.x and N7.y variants). Each point represents the median activity of promoter variants measured in the same nucleosomal context. Data are derived from Qi et al.^13^. (**D-E**) Effect of biological context on prediction accuracy under replicate-level and sequence-disjoint evaluations. Explained variance (R²) is shown in **D** and prediction error (MSE) in **E**. Context and motif-level features could be incorporated into the model framework, but did not uniformly improve accuracy under the stricter sequence-disjoint evaluation. **(F)** Conceptual summary of promoter regulation. DNA sequence, including motif grammar, defines baseline promoter activity, which is strongly modulated by hormonal input and fine-tuned by − 1/+1 nucleosomal sequences.

While the core promoter sequence provides a strong baseline for expression, biological context including hormonal signalling and chromatin environment can modulate expression levels. As previously established^13^, developmental and constitutive promoters differ markedly in their responsiveness to hormonal activation, while nucleosomal sequences flanking the core promoter exert an additional, though weaker, effect on expression. Through statistical testing we showed that developmental promoters show significantly higher ecdysone inducibility than constitutive promoters (Fig. 4A-B; Wilcoxon rank-sum test, *p* = 0.0054), whereas − 1/+1 nucleosomal sequences modulate expression levels more moderately, and in a promoter-dependent manner (Fig. 4C).

To examine whether promoter sequence and biological context could be represented within a single predictive framework, we extended the DNABERT-based model to incorporate both types of information. In addition to the core promoter sequence, the model included the Ecd activation state and quantitative features describing the − 1 and + 1 nucleosomal flanking sequences. We used quantitative context features rather than raw flanking sequences because the dataset is relatively small and contains only a limited number of distinct nucleosomal sequence contexts. Directly including long flanking sequences could therefore increase the risk of overfitting or memorization of the available contexts. The context features were concatenated with the sequence embedding and passed through additional fully connected layers before regression (Methods).

In the replicate-level split, all models showed high predictive performance (Fig. 4D, E and Supplementary Fig. 3). The sequence-only model achieved R² = 0.91 and MSE = 0.70. The sequence + context model retained similar explanatory power, with R² = 0.89 and MSE = 1.43, and the sequence + context + motif-feature model achieved R² = 0.91 and MSE = 1.16. Thus, the additional biological context and motif-level features could be integrated into the DNABERT-based framework while preserving high replicate-level predictive accuracy.

We then evaluated the same models using the stricter sequence-disjoint split, in which promoter sequences in the test set were not represented in the training set. Under this more conservative evaluation, all models showed lower performance, as expected. The sequence-only model achieved R² = 0.64 and MSE = 2.51, the sequence + context model achieved R² = 0.63 and MSE = 3.69, and the sequence + context + motif-feature model achieved R² = 0.60 and MSE = 4.00. Although the addition of context and motif features did not improve accuracy under this stricter split, the integrated models still retained substantial predictive signal, with R² values close to the sequence-only model.

Together, these results show that promoter sequence, Ecd activation state, nucleosomal context features, and motif-level information can be combined within a single DNA language model-based framework. The main value of this analysis is therefore the successful integration of heterogeneous biological features without loss of the overall predictive relationship, rather than a consistent improvement in accuracy in the current dataset. Larger datasets with more diverse nucleosomal contexts will be needed to determine whether context information, including raw flanking sequences, can improve generalization to unseen chromatin contexts.

### Gene-wise cross-validation reveals promoter class-specific generalization

A key question is whether the model can generalize to promoters from genes that were not seen during training. To address this, we performed gene-wise cross-validation, in which all promoter variants from a given gene were held out as the validation set, and the model was trained from scratch on promoters from all remaining genes. This procedure prevents information leakage and directly tests generalization across genes^17^.

**Fig. 5:**
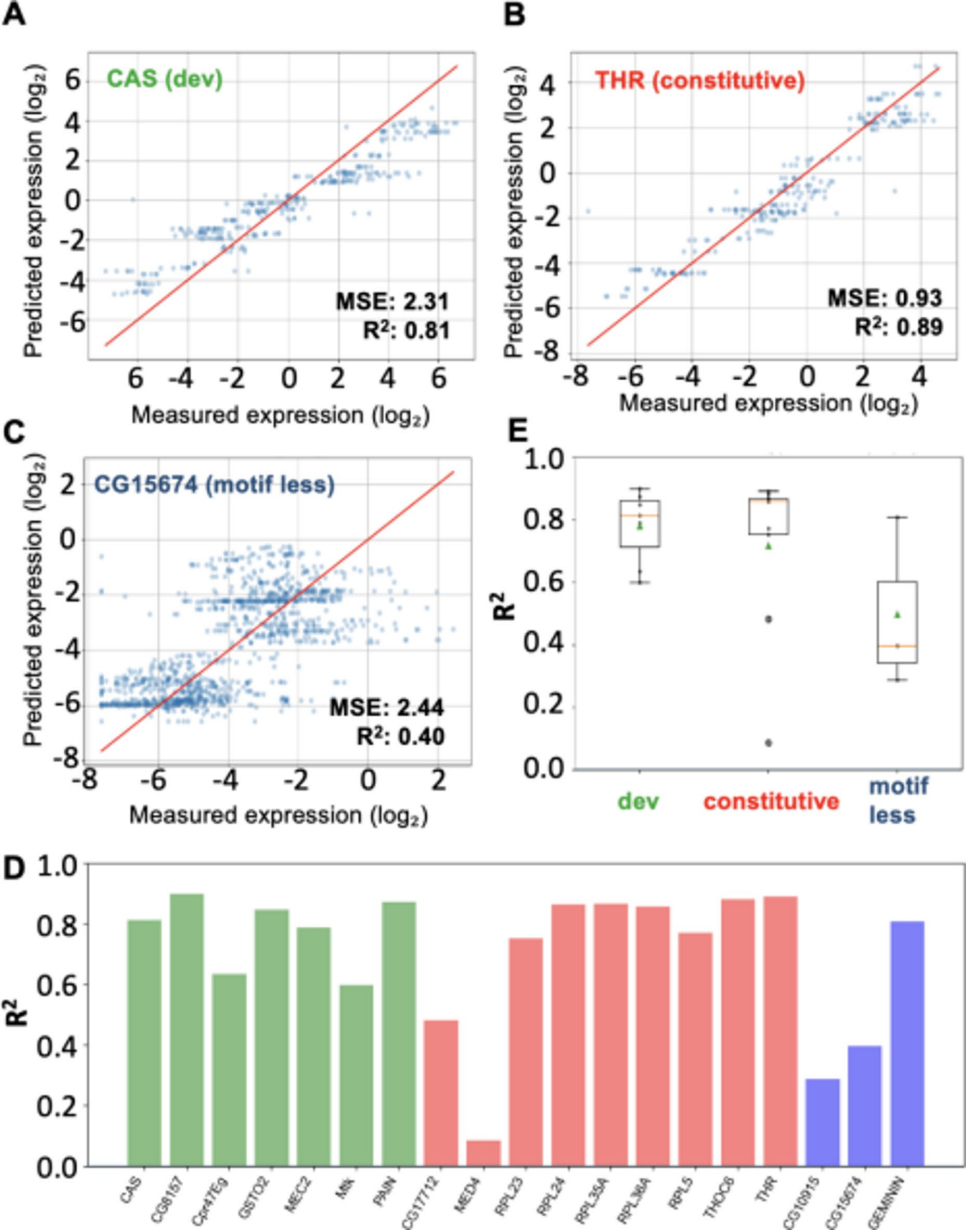
Gene-wise held-out validation reveals promoter-specific differences in generalization. (**A–C**) Predicted versus measured promoter activity for representative held-out genes. Shown are examples for the developmental promoter CAS (**A**), the constitutive promoter THR (**B**), and the motif-less promoter CG15674 (**C**). For each analysis, all promoter variants and replicate measurements from the indicated gene were excluded from training and used only for testing. (**D**) Gene-wise prediction performance when all promoter variants from a given gene are excluded from training. Each bar represents one held-out gene and is coloured by promoter class. (**E**) Distribution of gene-wise prediction performance grouped by promoter class. Boxes indicate median and interquartile range; whiskers show the full data range. Developmental and constitutive promoters showed slightly higher median R² values than motif-less promoters, but the difference between promoter classes was not statistically significant (one-way ANOVA, F = 1.67, *p* = 0.22).

To explore this, we tested three representative examples: CAS, THR and CG15674. For the developmental promoter CAS, predicted and measured activities showed good agreement (MSE = 2.31, R² = 0.81; Fig. 5A). Similarly strong performance was observed for the constitutive promoter THR (MSE = 0.93, R² = 0.89; Fig. 5B). In contrast, predictions for the motif-less promoter CG15674 were more variable (MSE = 2.44, R² = 0.40; Fig. 5C), illustrating that gene-wise performance can differ between individual promoters.

We next evaluated prediction performance across all investigated genes. Gene-wise performance was generally high (Fig. 5D and Supplementary Fig. 4), with most promoters showing substantial agreement between predicted and measured activity. Developmental and constitutive promoters such as RpL24, RpL35A, RpL36A, Thoc6, and THR were consistently well predicted (R² typically ~ 0.6–0.9; Fig. 5D). In contrast, some promoters, including MED4 and CG17712, showed lower predictive performance. Among motif-less promoters, performance ranged from relatively low (e.g., CG10915 and CG15674) to high (e.g., GEMININ, R² = 0.81), indicating heterogeneity within this group.

To assess whether prediction accuracy differed systematically across promoter classes, we compared gene-wise R² distributions for developmental, constitutive, and motif-less promoters (Fig. 5E). Developmental and constitutive promoters showed slightly higher median R² values than motif-less promoters. However, these differences were modest and not statistically significant (one-way ANOVA comparing mean R² across the three classes: F = 1.67, *p* = 0.22). These results suggest that generalization performance is influenced more by individual gene characteristics than by promoter class alone.

We further tested whether gene-wise performance depended on promoter strength, TATA status, motif richness, motif count, GC content, or the size of the training set (Supplementary Fig. 5). Prediction performance was broadly similar across weak, intermediate, and strong promoters, with no significant difference between groups (one-way ANOVA, *p* = 0.751) (Supplementary Fig. 5 A). TATA-containing promoters showed slightly higher average performance than TATA-less promoters, but this difference was also not significant (Welch’s t-test, *p* = 0.446) (Supplementary Fig. 5B). In contrast, motif-richness showed the clearest association with gene-wise performance (Supplementary Fig. 5 C): motif-poor promoters had lower and more variable validation R² values than motif-rich promoters, and this group difference was significant (one-way ANOVA, *p* = 0.0314). Consistent with this observation, the number of annotated motifs per promoter showed a moderate positive trend with validation R² (Supplementary Fig. 5D), although this continuous correlation did not reach statistical significance (Pearson *r* = 0.43, *p* = 0.0634; Spearman ρ = 0.42, *p* = 0.0735). GC content was not significantly correlated with validation R² (Pearson *r* = − 0.13, *p* = 0.631; Spearman ρ = −0.31, *p* = 0.201) (Supplementary Fig. 5E). Training set size also did not significantly explain gene-wise variability (Pearson *r* = 0.32, *p* = 0.186; Spearman ρ = 0.39, *p* = 0.102; Supplementary Fig. 5 F). Together, these analyses suggest that motif richness may contribute to differences in gene-wise prediction performance, whereas promoter strength, TATA status, GC content and training-set size showed no significant association in this analysis.

### Generalization to in vivo Drosophila embryo promoter activity

**Fig. 6:**
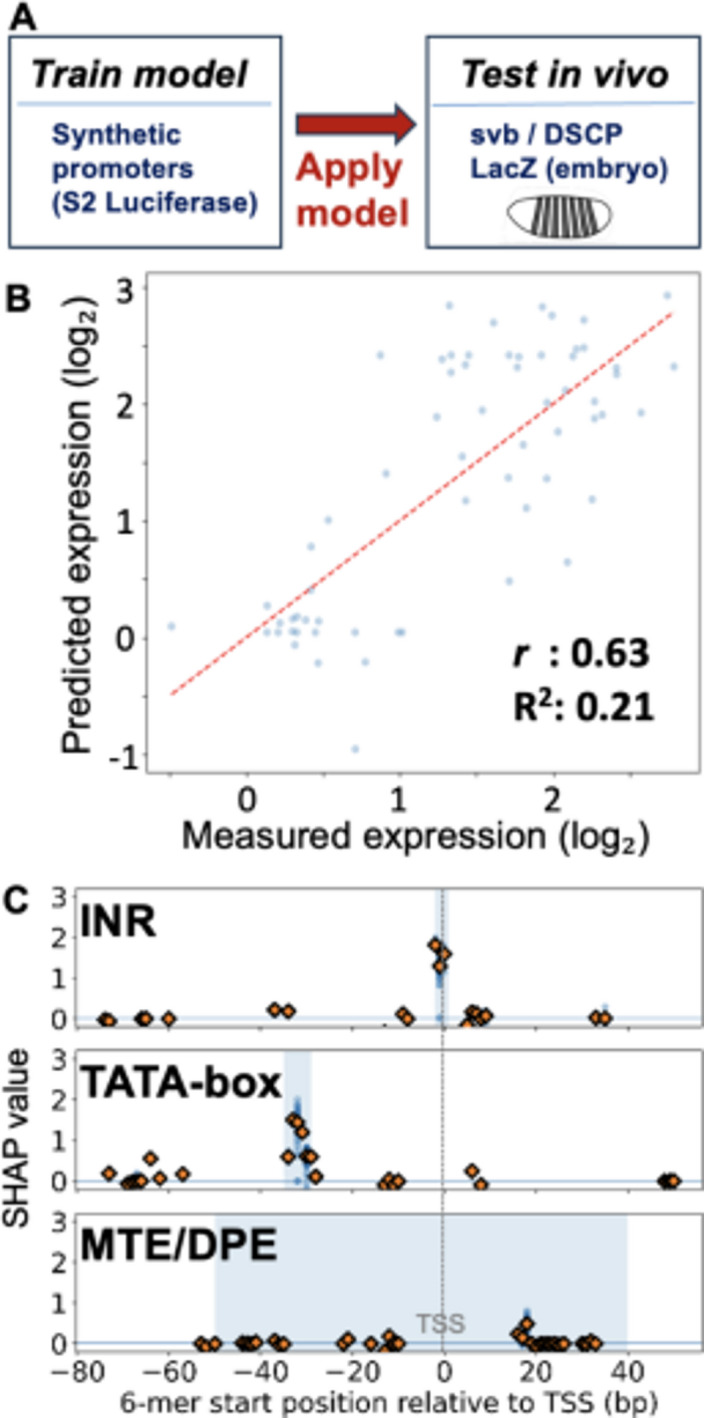
Generalization to in vivo *Drosophila* embryo promoter activity. **(A)** Schematic illustrating the validation strategy of model performance on an independent *Drosophila* embryo dataset. A model trained exclusively on synthetic promoter activity measured in S2 cells is applied without retraining to promoter sequences from an independent in vivo embryo reporter assay^29^.(**B**) Scatter plot showing Pearson correlation (r) and coefficient of determination (R²) between promoter activity predicted by our model and measured activity in *Drosophila* embryos. (**C**) SHAP analysis applied to the in vivo embryo promoter variants. Blue shaded regions mark motif genomic regions previously identified by the bioinformatic analysis of Qi et al.^13^ High model-attribution 6-mers overlap sequence features corresponding to known core promoter elements, including INR, TATA-box, and MTE/DPE-related positions, with the strongest attributions located at expected positions relative to the TSS.

Finally, to test generalization beyond the synthetic in vitro system, we evaluated our model on an independent in vivo dataset from Li and colleagues^29^, which measured the activity of randomly mutated developmental promoters (svb and DSCP) in Drosophila embryos (Fig. 6A). This dataset provides a challenging test, as *transcription in vivo* occurs in a complex regulatory environment that includes enhancer-promoter interactions, chromatin state, transcription factor availability, and developmental stage.

We report R² for consistency with previous analyses and additionally provide Pearson correlation (r) to quantify the strength of the linear relationship between predicted and measured values. The model, trained on luciferase promoter measurements and without retraining or parameter adjustment, achieved partial but limited performance (Pearson *r* ≈ 0.63; R² ≈ 0.21; Fig. 6B), capturing overall trends in promoter activity but explaining only part of the quantitative variance. Predictions showed partial separation consistent with the two underlying promoter backbones in the dataset, reflecting differences in baseline activity.

The model performance in embryos was lower than in S2 cells, consistent with additional regulatory layers in vivo, including enhancer-promoter interactions^24^, chromatin accessibility^24^, and developmental timing^17^ factors that are not encoded in the core promoter sequence. Notably, this level of performance is comparable to that observed for some individual promoters in the gene-wise cross-validation analysis (e.g., CG10915 or MED4; Fig. 5), suggesting that reduced accuracy may arise from a combination of promoter-specific characteristics and the additional regulatory complexity present in vivo.

We also applied SHAP to the embryo promoter variants to examine whether the model assigned high importance to biologically interpretable positions (Fig. 6C). High-SHAP positions overlapped regions corresponding to known core promoter elements, including INR, TATA-box, and MTE/DPE-related positions, with the strongest signal located at expected positions relative to the TSS. The MTE/DPE region was considered because it corresponds to a motif region identified by the genomic bioinformatic analysis of Qi et al.^13^However, our luciferase assay measurements indicate that this motif has only weak functional activity in the tested promoter context^13^. Consistent with this experimental observation, the SHAP signal at the MTE/DPE-related positions was weaker than the strongest INR- and TATA-associated attributions.

Because the model uses overlapping 6-mer tokens, SHAP values cannot be assigned unambiguously to individual single-nucleotide mutations. A single base substitution can affect up to six neighbouring 6-mer tokens. We therefore interpreted the embryo SHAP analysis at the level of motif-associated 6-mers and promoter regions, rather than as direct single-nucleotide mutation attribution. Thus, Fig. 6C identifies promoter regions that contribute to the model prediction, but it does not show that each individual mutation is independently important or that individual mutations alone determine the in vivo expression output.

Together, these results indicate that core promoter sequence captures part of the transcriptional output in vivo, but does not fully explain expression in a complex developmental setting. The embryo analysis shows that the model can partly generalize to a different biological context while still highlighting meaningful promoter features. However, it does not support broad generalization across all cell types or developmental stages.

## Discussion

Our study shows that DNA language models can learn sequence-expression relationships from a small, controlled *Drosophila* synthetic promoter library, but the estimated accuracy depends strongly on the evaluation design. The original replicate-level split yielded high apparent performance (R² ≈ 0.91; MSE ≈ 0.70), whereas the stricter sequence-disjoint split yielded lower but still meaningful predictive signal (R² ≈ 0.64; MSE ≈ 2.51). This decrease is expected because the sequence-disjoint split tests prediction of promoter sequences not present during training, rather than new replicate measurements of sequences already represented in the training data.

The interpretability analyses suggest that the model learned promoter features that are consistent with known core promoter architecture. SHAP analysis revealed contributions from canonical promoter elements, including the INR, TATA box, MTE/DPE, DRE, and Ohler motifs, consistent with prior experimental studies. The positional SHAP analysis also showed that important motif-related 6-mers appear at expected positions relative to the TSS. These findings support biological interpretability, but SHAP remains a model explanation method: it identifies features that influence the model prediction and does not by itself establish direct causality.

The context-aware models show that additional biological features can be incorporated into the DNABERT-based framework. In these models, promoter sequence embeddings were combined with ecdysone activation state and quantitative features describing the − 1 and + 1 nucleosomal flanking regions. The integrated models retained strong performance in the replicate-level split and maintained substantial predictive signal under the stricter sequence-disjoint split. However, adding context features did not consistently improve accuracy compared with the sequence-only model. We therefore interpret these results as a proof of concept that heterogeneous biological information can be represented within a single model, rather than as evidence that the current context features uniformly improve prediction. A likely limitation is the small size of the dataset and the limited diversity of nucleosomal sequence contexts. Future studies with larger libraries and more diverse flanking contexts will be needed to test whether raw flanking sequences or richer chromatin features can improve prediction without overfitting.

Validation on the independent *Drosophila* embryo dataset showed partial but limited generalization. The model trained on S2-cell luciferase measurements captured some of the in vivo transcriptional output, but it did not fully explain expression in the embryo. This is expected, because embryo expression depends on regulatory information beyond the core promoter sequence, including enhancer-promoter interactions, chromatin accessibility, transcription factor availability, developmental stage, and cell-type-specific regulatory environments. The embryo analysis therefore shows that the model can partly generalize to a different biological context and still highlight meaningful promoter features, but it does not support broad generalization across all cell types or developmental stages.

These results place our study in the context of recent promoter-focused deep learning approaches such as PARM, Puffin, and PromoterAI, which use different architectures and training objectives to model human promoter activity, transcription initiation, or promoter-variant effects^18,19^.–^20^ In contrast, our study focuses on a pretrained DNA language model fine-tuned on a small, controlled *Drosophila* core-promoter library. This setting allowed us to evaluate both prediction and interpretation at the level of promoter motifs and 6-mer tokens. The comparison with a 6-mer ridge model and a simple CNN suggests that much of the predictive signal in this dataset is local. DNABERT-2 did not clearly outperform the simple CNN under the stricter sequence-disjoint evaluation. Thus, the main value of the DNABERT-2 framework in this study is not only prediction accuracy, but also its ability to provide interpretable 6-mer-level attributions that can be related to known core promoter elements.

Several limitations should be considered. First, the dataset is small relative to the size of the pretrained model, which limits the ability to evaluate generalization to diverse promoter architectures. Second, the synthetic library provides a controlled setting, but it cannot fully capture the regulatory complexity of endogenous genomic *loci*. Third, the model currently produces point predictions without uncertainty estimates. Future work should incorporate uncertainty estimation, for example through model ensembles or Bayesian approaches, to identify predictions that are less reliable, especially for out-of-distribution promoter sequences. Larger datasets that combine systematic promoter perturbations, chromatin context, enhancer information, and in vivo measurements will be needed to build models that generalize more broadly across regulatory settings.

Overall, our results show that transformer-based DNA language models can predict promoter activity in controlled settings and identify sequence features that match known promoter motifs and their expected positions. These findings are useful for forming hypotheses about promoter architecture and for guiding future promoter engineering.

## Methods

### Synthetic promoter dataset

We used the synthetic promoter dataset generated in our previous work^13^, which systematically perturbs *Drosophila *core promoter sequences and measures their activity using a dual-luciferase reporter assay in S2 cells^30^. Each construct consists of a − 1 nucleosomal sequence (N1.x), an enhancer module, a 131 bp core promoter region centered on the transcription start site (TSS), and a + 1 nucleosomal sequence (N7.y)^13^. Promoter activity was quantified as Firefly/Renilla luciferase ratios and normalized across experiments.

For the base sequence-only model, we restricted the dataset to promoter variants measured without ecdysone induction and flanked by fixed − 1 and + 1 nucleosomal sequences (N1.x and N7.y)^13^. Expression values were log₂-transformed after clipping low values (minimum 5 × 10⁻⁴) to ensure numerical stability. Unless otherwise stated, individual biological replicates were treated as separate measurements during replicate-level model training to preserve experimental variation. For analyses requiring a single expression value per promoter sequence, including motif expression strength and promoter-level summaries, replicate measurements were summarized by the median expression value for each promoter sequence.

### Sequence preprocessing and k-mer tokenization

Promoter sequences were trimmed to the 131 bp core promoter region. Sequences were tokenized using overlapping fixed-length k-mers (k = 6, stride = 1), yielding 126 k-mer tokens per sequence. Following the DNABERT-2 input convention, special [CLS] and [SEP] tokens were added.

Although DNABERT-2 natively supports subword-based tokenization, we empirically found that fixed 6-mer tokenization provided comparable predictive performance while enabling substantially improved interpretability. Six-mers closely match the length scale of canonical core promoter motifs and allowed stable attribution of sequence features using SHAP.

### Model architecture and fine-tuning

We fine-tuned the pre-trained DNABERT-2 (117 M parameters) transformer model for regression by replacing the classification head with a single linear output neuron. Model weights were initialized from the publicly available DNABERT-2 checkpoint.

Models were trained using mean squared error (MSE) loss and optimized with *AdamW*. Unless otherwise noted, hyperparameters were: learning rate 1 × 10⁻⁵, batch size 28, weight decay 4–6 × 10⁻⁴, and 25 training epochs. Dropout probabilities in both hidden and attention layers were set to very low values (≈ 1 × 10⁻⁵) to stabilize regression. Training was performed on NVIDIA GPUs (GeForce GTX 1080 or equivalent). Training typically required 30–120 min per model.

### Train–test splitting and gene-wise cross-validation strategies

For standard replicate-level evaluation, promoter measurements were randomly split into 90% training and 10% held-out test sets. Because this split can place biological replicates of the same promoter sequence in both training and test sets, we added a sequence-disjoint split in which all measurements from the same promoter sequence were assigned together to either training or test data. This prevents exact promoter-sequence overlap between training and test sets. To assess generalization beyond individual genes, we additionally performed gene-wise cross-validation, in which all promoter variants derived from a given gene were held out entirely as the test set, and a new model was trained from scratch using promoter variants from all other genes. This process was repeated for each gene, ensuring strict separation between training and validation data. Model performance was evaluated using mean squared error (MSE), coefficient of determination (R²), and Pearson correlation coefficient.

Baseline models were evaluated under the same sequence-disjoint split. For the 6-mer ridge regression baseline, each promoter was represented by its 6-mer content and a ridge regression model was trained to predict log₂ promoter activity. For the CNN baseline, promoter sequences were one-hot encoded and used as input to a simple convolutional neural network trained for regression. The CNN was used as a prediction baseline rather than as a second primary interpretation model, because its one-hot nucleotide input yields base-level features, whereas DNABERT-2 6-mer tokens allow SHAP attributions to be linked more directly to known promoter motifs.

### Context-aware models

To incorporate biological context beyond core promoter sequence, we extended the base DNABERT-2 model to include additional inputs: (i) a binary indicator of Ecd induction, and (ii) quantitative features representing the − 1 and + 1 nucleosomal context (N1.x and N7.y)^13^. These features were concatenated with the DNABERT-derived sequence representation and processed through two additional fully connected layers prior to regression. We used quantitative context features rather than raw flanking sequences because the dataset contains relatively few distinct nucleosomal contexts; including long flanking sequences directly could increase input length, model complexity, and the risk of memorizing the available contexts rather than learning generalizable flanking-sequence rules.

In a further extension, explicit motif-derived features were included. These comprised PWM-based motif scores and positional information for a curated set of known *Drosophila* core promoter motifs. Motif features were standardized using z-score normalization based on the training set only. While this model yielded marginal performance gains, the increased complexity led us to use the sequence + context model as the primary architecture.

### Model interpretability and feature attribution

To interpret sequence-based predictions, we applied SHapley Additive exPlanations (SHAP)^25^ at the k-mer level. The SHAP analyses were performed using the replicate-level trained sequence-only model and were intended for biological interpretation and model probing, not as an estimate of sequence-disjoint generalization. SHAP values were computed using a background distribution of randomly generated DNA sequences matched in length to the input promoters. For each sequence, SHAP attribution scores were assigned to individual 6-mer tokens, indicating their contribution to predicted expression. We used SHAP as the primary interpretation method because its additive 6-mer-level scores can be compared across promoters and related to motif-like sequence tokens. We did not add LIME as a parallel analysis because local perturbation of overlapping 6-mers can alter neighbouring sequence context and may be more difficult to interpret.

SHAP values were aggregated across sequences to identify globally informative k-mers and to compare their distributions and positional patterns across promoter classes. For selected promoters, SHAP scores were visualized along the sequence and compared with independently identified motif annotations. SHAP analyses were also applied to the independent embryo promoter dataset to test whether high-attribution positions in mutated promoters corresponded to biologically interpretable core promoter elements.

To associate SHAP-enriched 6-mers with known core promoter elements, we compared high-impact 6-mers to consensus motifs identified previously using *XXmotif*^13^. This mapping was performed manually by sequence comparison, allowing up to two single-nucleotide mismatches relative to the *XXmotif*-derived consensus sequences. This approach enabled approximate assignment of SHAP-enriched k-mers to canonical promoter motifs while accounting for motif degeneracy and local sequence variability.

### Attention analysis

To complement SHAP-based interpretation, attention weights were extracted from all transformer layers and heads during inference. Attention maps were visualized for individual promoters and averaged across layers, heads, and sequences to identify consistently attended regions. As attention patterns were highly distributed and complex, these analyses were used qualitatively and reported in Supplementary Fig. 2.

### Analysis of gene-wise performance determinants

To examine factors associated with gene-wise prediction performance, held-out genes were grouped according to promoter strength, motif richness, TATA-box status, GC content, and training-set size. Promoter strength was defined from the median log₂ promoter activity of all variants belonging to each gene, and genes were divided into weak, intermediate, and strong groups using tertiles of this distribution. Motif richness was defined as the number of annotated core promoter motifs per gene, based on the *XXmotif*-derived motif annotations from Qi et al.; genes were divided into motif-poor, intermediate, and motif-rich groups using tertiles of the motif-count distribution. TATA status was assigned according to whether a TATA-box motif was annotated in the corresponding promoter. GC content was calculated as the fraction of G and C nucleotides in the 131 bp core promoter sequence. For each gene-wise held-out model, training-set size was counted as the number of promoter activity measurements remaining after excluding all variants and replicate measurements from the held-out gene. Differences in validation R² between promoter-strength and motif-richness groups were tested using one-way ANOVA. Differences between TATA-containing and TATA-less promoters were tested using Welch’s t-test. Associations between validation R² and continuous variables, including motif count, GC content, and training-set size, were assessed using Pearson and Spearman correlation analyses.

### Prediction for the in vivo validation

External validation used published in vivo embryo reporter data from Li et al.^29^, measuring the activity of mutated svb and DSCP promoters. To ensure compatibility with the DNABERT-2 model, we aligned the transcription start site (TSS) of each Li et al. sequence to the TSS and extracted a 131 bp window centered on the TSS, matching the core promoter length used throughout this study. The DNABERT-2 model trained on S2 cell data was applied directly to these cropped sequences without retraining or parameter tuning.

Predicted promoter activities were compared to the corresponding measured expression values to evaluate generalization across experimental systems. We additionally computed SHAP values for the embryo promoter variants to determine whether model-attributed high-importance positions coincided with known core promoter elements and mutation-sensitive regions. Because the model input consisted of overlapping 6-mer tokens, embryo SHAP values were interpreted at the level of mutation-overlapping or motif-associated 6-mer regions, rather than as unambiguous single-nucleotide mutation attributions. These in vivo SHAP results were interpreted as model-based attribution rather than experimental evidence of causality.

### Software and reproducibility

All analyses were performed in *Python* using *PyTorch* and the *HuggingFace* Transformers library. Custom scripts were used for model training, prediction, interpretability analyses, and visualization. Model configurations, training parameters, and evaluation outputs were logged and exported to enable reproducibility.

## Supplementary Information

Below is the link to the electronic supplementary material.

Supplementary Material 1

## Data Availability

All experimental datasets analyzed in this study have been published previously. Synthetic promoter activity data measured in *Drosophila* S2 cells are available from Qi *et al.* (2022) (ref13). In vivo embryo promoter activity data were obtained from Li *et al.* (2023) (ref 29).

## Code availibility

All custom code and data used for model training, evaluation, and interpretability analyses were made publicly available on *GitHub* (https://github.com/GeneCenterMunich/decoding_promoter_activity).
