# Supplementary material for: Decoding promoter activity from DNA sequence using pre-trained language models
Source: Sci Rep. 2026 Jul 14;16:22050. doi: 10.1038/s41598-026-61483-w (PMC13369180; doi:10.1038/s41598-026-61483-w)
Supplement: Supplementary file 1 — Supplementary Material 1 [file 41598_2026_61483_MOESM1_ESM.pdf]

SUPPLEMENTARY INFORMATION

FOR

Decoding Promoter Activity from DNA Sequence using Pre-trained Language Models

Christophe Jung

Gene Center and Department of Biochemistry, Quantitative and Molecular Biology (QMB), Ludwig-Maximilians-Universität München, Feodor-Lynen-Strasse 25, 81377 München, Germany

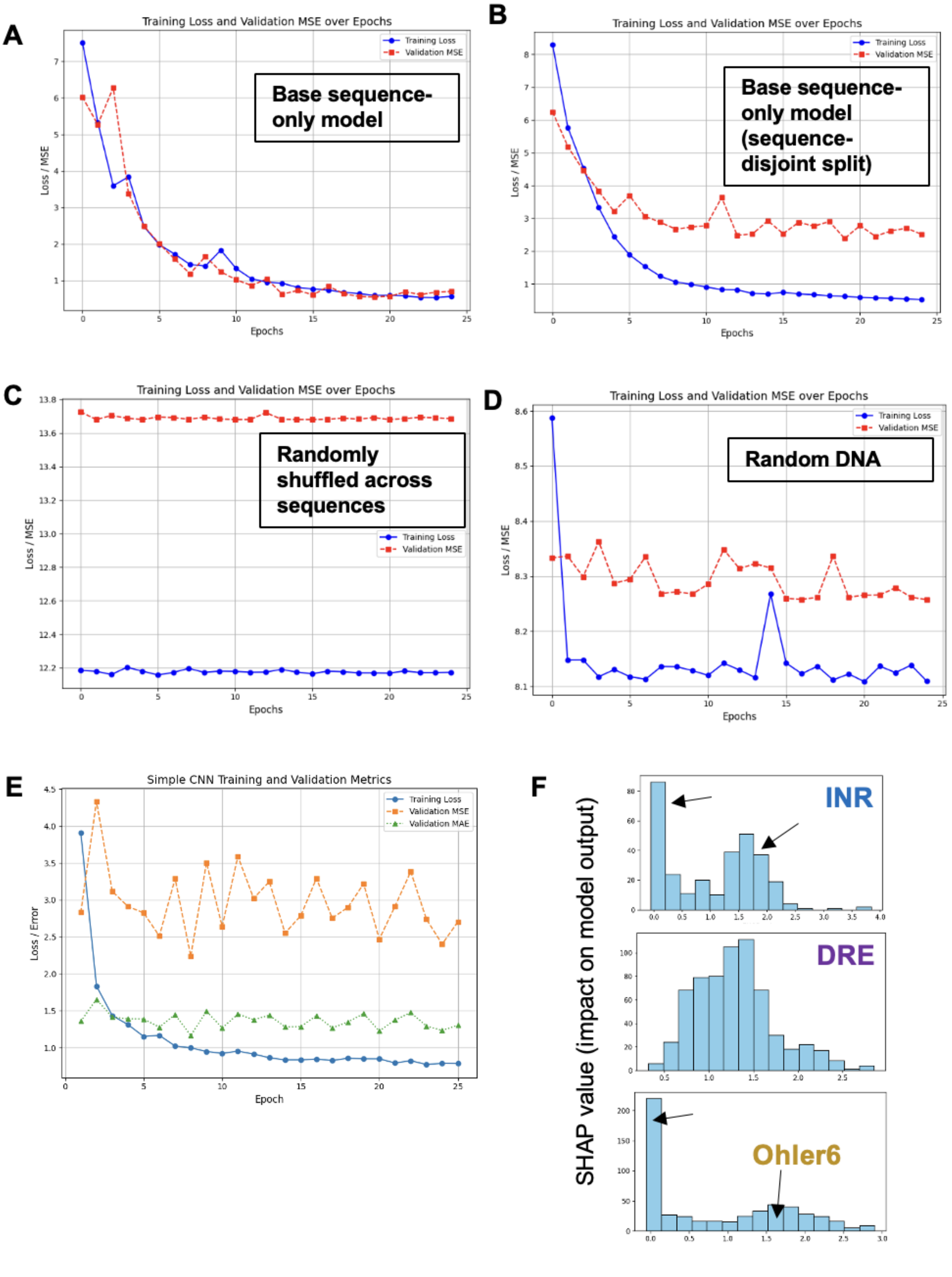
A
Training Loss and Validation MSE over Epochs
Base sequence-only model
Training Loss
Validation MSE
Loss / MSE
Epochs
B
Training Loss and Validation MSE over Epochs
Base sequence-only model (sequence-disjoint split)
Training Loss
Validation MSE
Loss / MSE
Epochs
C
Training Loss and Validation MSE over Epochs
Randomly shuffled across sequences
Training Loss
Validation MSE
Loss / MSE
Epochs
D
Training Loss and Validation MSE over Epochs
Random DNA
Training Loss
Validation MSE
Loss / MSE
Epochs
E
Simple CNN Training and Validation Metrics
Training Loss
Validation MSE
Validation MAE
Loss / Error
Epoch
F
SHAP value (impact on model output)
INR
DRE
Ohler6

Supplementary Figure 1. Model training stability and controls

Validation loss curves and control analyses.

(**A**) Base sequence-only model under the replicate-level split.

(**B**) Base sequence-only model under the sequence-disjoint split.

(**C**) Model fine-tuned after randomly shuffling expression labels across sequences (Methods). The relationship between promoter sequence and measured activity was disrupted by assigning expression values to the wrong sequences. Both training loss and validation MSE remained high and nearly unchanged across epochs, indicating that the model could not learn a meaningful sequence-activity relationship after label shuffling.

(**D**) Model trained after replacing promoter sequences with random DNA of matched length and composition (Methods). Training loss decreased slightly, but validation MSE remained high and largely unchanged across epochs, indicating that random DNA does not contain a generalizable sequence signal for promoter activity prediction.

(**E**) Simple CNN baseline model. The CNN showed decreasing training loss, but validation MSE and MAE remained high and variable across epochs. This indicates limited generalization to held-out data.

(**F**) Distributions of SHAP values for representative motif classes. Initiator-associated 6-mers show a bimodal distribution (upper panel), DRE-associated 6-mers display a broad distribution (middle panel), and TTGTT/Ohler6-associated 6-mers show overlapping bimodal distributions, reflecting differences in positional constraints and regulatory roles (lower panel).

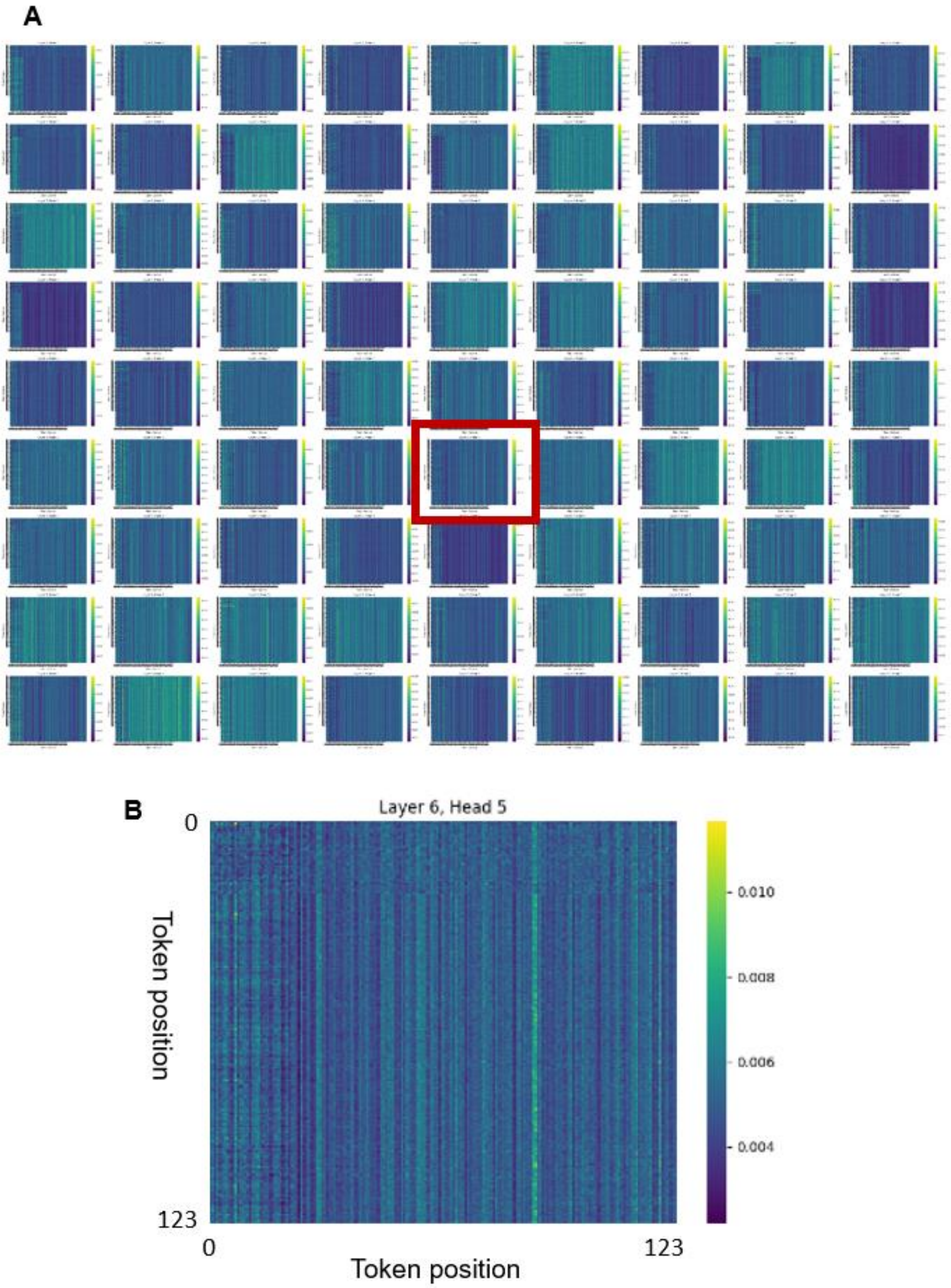
A
B
Layer 6, Head 5
0
123
Token position
0
123
Token position
0.010
0.008
0.006
0.004

Supplementary Figure 2. Attention map visualizations

(A) Representative attention maps from the transformer model. DNABERT-2 contains 12 transformer layers with 12 attention heads each, yielding 144 attention matrices per sequence, each of size ~130×130. While averaged attention highlighted regions near the TSS (Token position ~80) and downstream elements, the complexity of these patterns limited direct biological interpretation. The heatmap colour scale (right) indicates normalized attention weights, with higher values corresponding to stronger attention.

(B) Magnified view of the representative attention map (layer 6, head 5) indicated by the red rectangle in panel **A**. The colour scale is identical to that shown in panel **A**.

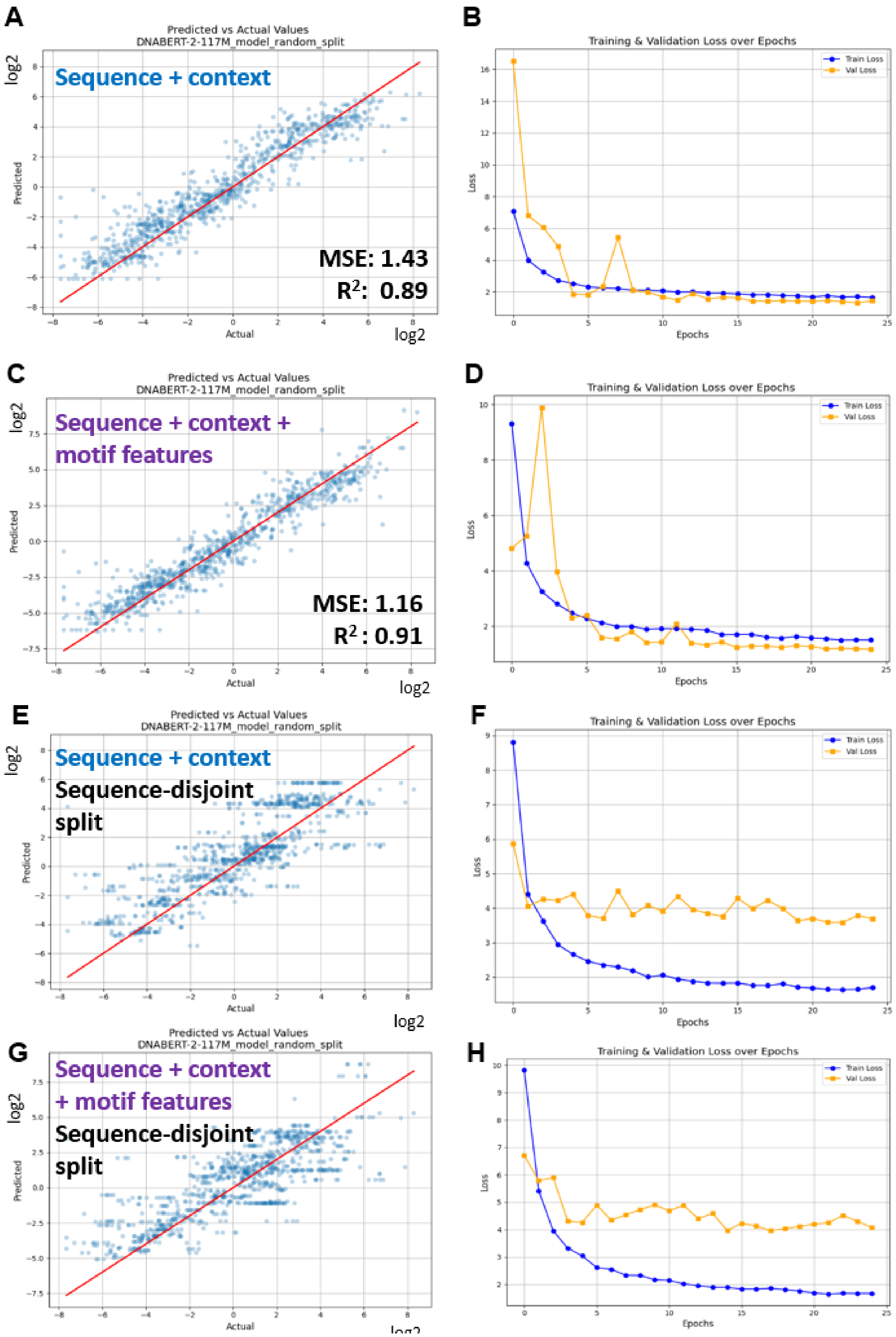
A
Predicted vs Actual Values
DNABERT-2-117M_model_random_split
Sequence + context
MSE: 1.43
R2: 0.89
log2
Predicted
Actual
B
Training & Validation Loss over Epochs
Train Loss
Val Loss
Loss
Epochs
C
Predicted vs Actual Values
DNABERT-2-117M_model_random_split
Sequence + context +
motif features
MSE: 1.16
R2: 0.91
log2
Predicted
Actual
D
Training & Validation Loss over Epochs
Train Loss
Val Loss
Loss
Epochs
E
Predicted vs Actual Values
DNABERT-2-117M_model_random_split
Sequence + context
Sequence-disjoint
split
log2
Predicted
Actual
F
Training & Validation Loss over Epochs
Train Loss
Val Loss
Loss
Epochs
G
Predicted vs Actual Values
DNABERT-2-117M_model_random_split
Sequence + context
+ motif features
Sequence-disjoint
split
log2
Predicted
Actual
H
Training & Validation Loss over Epochs
Train Loss
Val Loss
Loss
Epochs

Supplementary Figure 3. Performance of context-aware DNABERT-based models

Prediction performance and training curves for models integrating promoter sequence with biological context and motif-level features.

(**A**) Predicted versus measured promoter activity for the sequence + context model under the replicate-level split. The model showed high predictive performance, with MSE = 1.43 and R^2 = 0.89.

(**B**) Training and validation loss curves for the sequence + context model under the replicate-level split. Both losses decreased across epochs, indicating stable model training and good fit to the validation data.

(**C**) Predicted versus measured promoter activity for the sequence + context + motif-feature model under the replicate-level split. Adding motif-level features retained high predictive performance, with MSE = 1.16 and R^2 = 0.91.

(**D**) Training and validation loss curves for the sequence + context + motif-feature model under the replicate-level split.

(**E**) Predicted versus measured promoter activity for the sequence + context model under the sequence-disjoint split. Performance was lower than under the replicate-level split, reflecting the stricter evaluation in which test promoter sequences were not represented in the training set.

(**F**) Training and validation loss curves for the sequence + context model under the sequence-disjoint split. Training loss continued to decrease, whereas validation loss remained higher and more variable, indicating reduced generalization to unseen promoter sequences.

(**G**) Predicted versus measured promoter activity for the sequence + context + motif-feature model under the sequence-disjoint split.

(H) Training and validation loss curves for the sequence + context + motif-feature model under the sequence-disjoint split. The separation between training and validation loss indicates that generalization was more difficult under the sequence-disjoint evaluation.

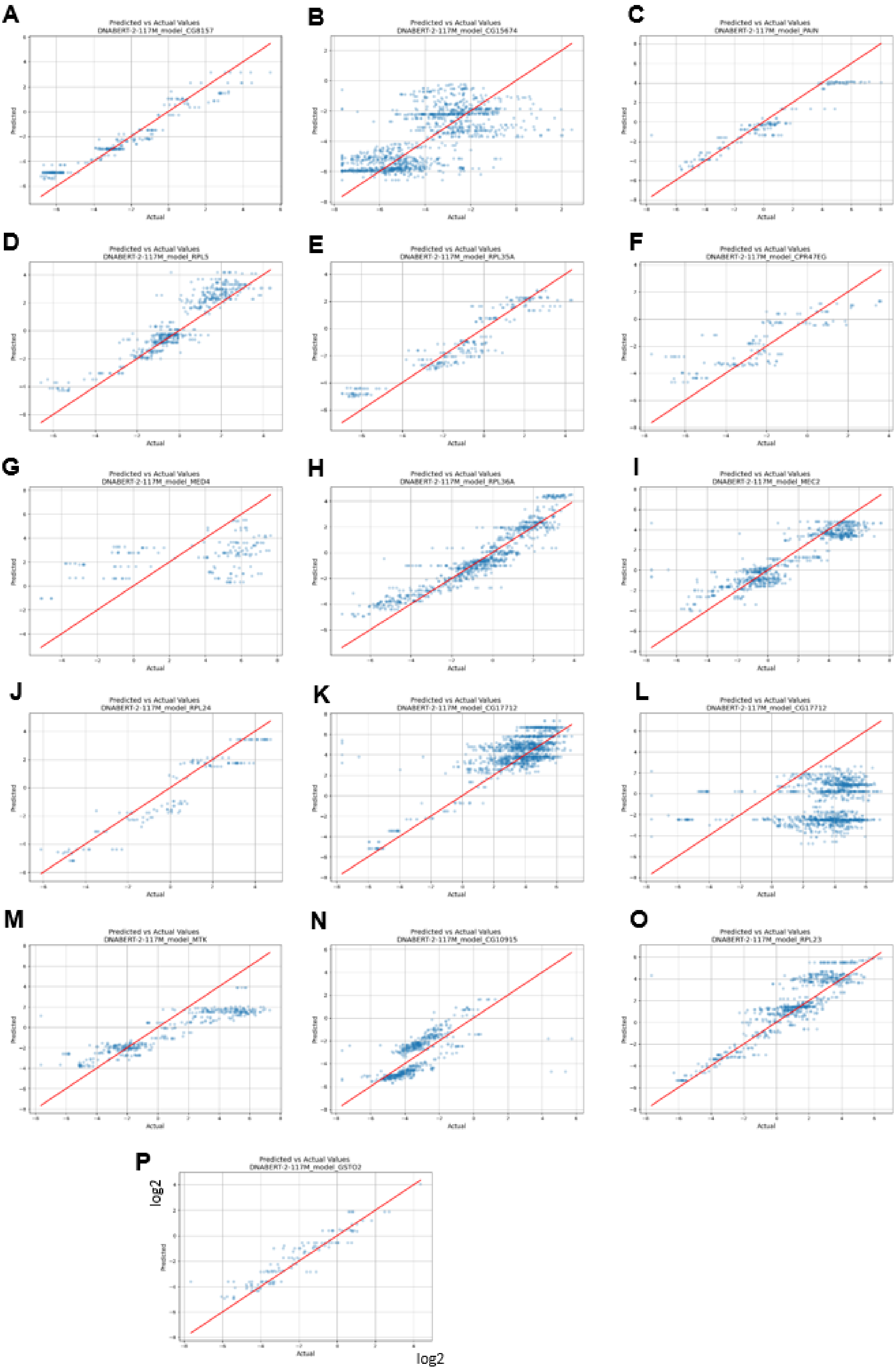
A
Predicted vs Actual Values
DNABERT-2-117M_model_CG8157
B
Predicted vs Actual Values
DNABERT-2-117M_model_CG15674
C
Predicted vs Actual Values
DNABERT-2-117M_model_PAIN
D
Predicted vs Actual Values
DNABERT-2-117M_model_RPL5
E
Predicted vs Actual Values
DNABERT-2-117M_model_RPL35A
F
Predicted vs Actual Values
DNABERT-2-117M_model_CPR47EG
G
Predicted vs Actual Values
DNABERT-2-117M_model_MED4
H
Predicted vs Actual Values
DNABERT-2-117M_model_RPL36A
I
Predicted vs Actual Values
DNABERT-2-117M_model_MEC2
J
Predicted vs Actual Values
DNABERT-2-117M_model_RPL24
K
Predicted vs Actual Values
DNABERT-2-117M_model_CG17712
L
Predicted vs Actual Values
DNABERT-2-117M_model_CG17712
M
Predicted vs Actual Values
DNABERT-2-117M_model_MTK
N
Predicted vs Actual Values
DNABERT-2-117M_model_CG10915
O
Predicted vs Actual Values
DNABERT-2-117M_model_RPL23
P
Predicted vs Actual Values
DNABERT-2-117M_model_GSTO2
Predicted
Actual
log2
log2

Supplementary Figure 4. Training dynamics for gene-wise held-out models

Training loss curves for the remaining gene-wise held-out models and additional gene-wise performance diagnostics. Most models show consistent convergence behavior, except for MED4 and CG10915, which display reduced predictive performance ($R^2 < 0.4$).

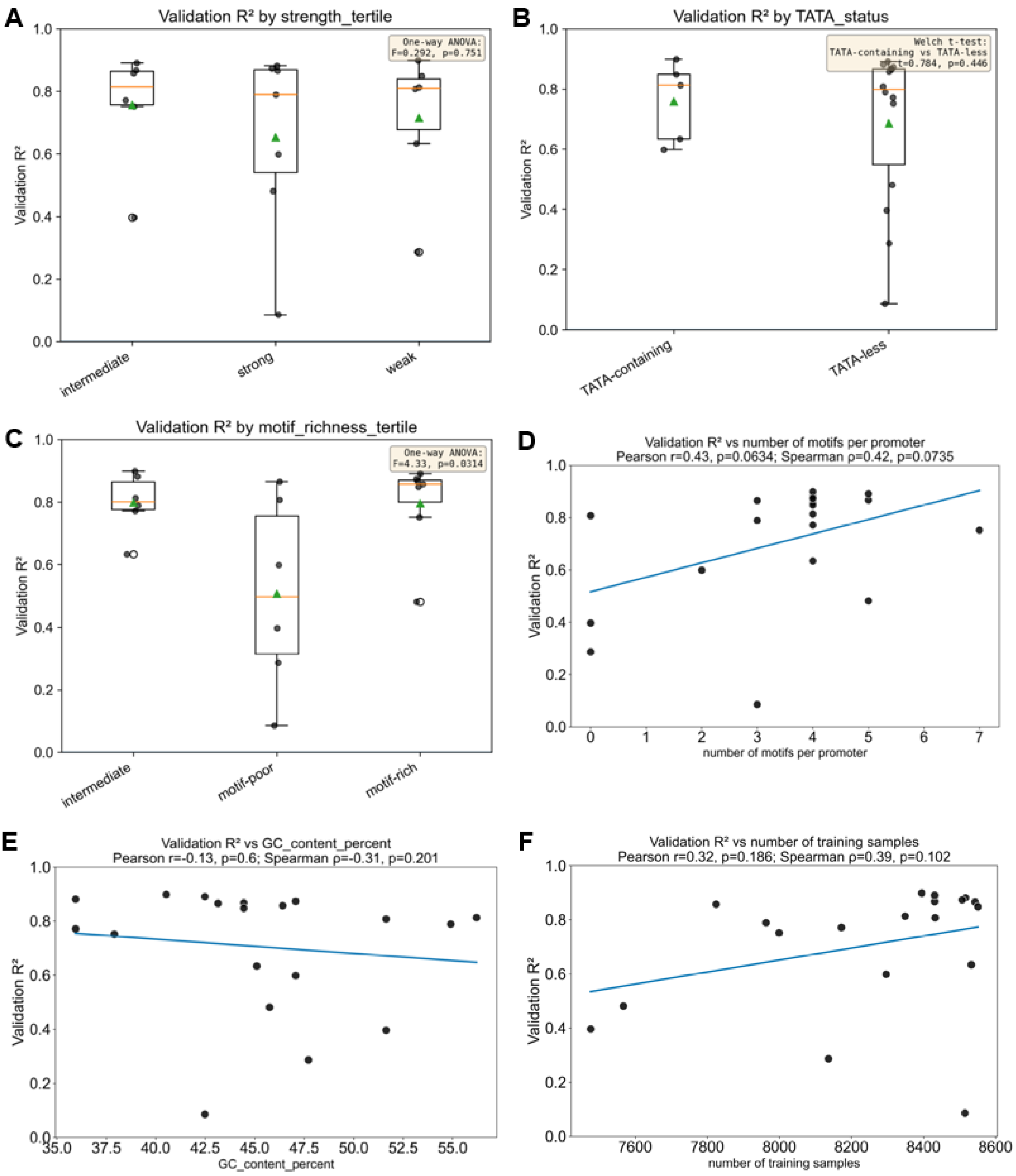
A
Validation R² by strength_tertile
One-way ANOVA:
F=0.292, p=0.751
Validation R²
intermediate
strong
weak
B
Validation R² by TATA_status
Welch t-test:
TATA-containing vs TATA-less
t=0.784, p=0.446
Validation R²
TATA-containing
TATA-less
C
Validation R² by motif_richness_tertile
One-way ANOVA:
F=4.33, p=0.0314
Validation R²
intermediate
motif-poor
motif-rich
D
Validation R² vs number of motifs per promoter
Pearson r=0.43, p=0.0634; Spearman ρ=0.42, p=0.0735
Validation R²
number of motifs per promoter
E
Validation R² vs GC_content_percent
Pearson r=-0.13, p=0.6; Spearman ρ=-0.31, p=0.201
Validation R²
GC_content_percent
F
Validation R² vs number of training samples
Pearson r=0.32, p=0.186; Spearman ρ=0.39, p=0.102
Validation R²
number of training samples

Supplementary Figure 5. Gene-wise prediction performance by promoter features.

Validation R^2 was analyzed across promoter groups defined by promoter strength, motif content, GC content, and training sample number.

(**A**) Validation R^2 grouped by promoter strength tertile. Model performance was similar across weak, intermediate, and strong promoters, with no significant difference between groups (one-way ANOVA, F = 0.292, p = 0.751).

(**B**) Validation R^2 grouped by TATA-box status. TATA-containing and TATA-less promoters showed no significant difference in validation performance (Welch t-test, t = 0.784, p = 0.446).

(**C**) Validation R^2 grouped by motif richness tertile. Motif-rich promoters showed higher and more stable validation performance than motif-poor promoters, with a significant difference between groups (one-way ANOVA, F = 4.33, p = 0.0314).

(**D**) Validation R^2 plotted against the number of motifs per promoter. A positive trend was observed, suggesting higher performance for promoters containing more annotated motifs, although the correlation did not reach conventional significance (Pearson r = 0.43, p = 0.0634; Spearman ρ = 0.42, p = 0.0735).

(**E**) Validation R^2 plotted against GC content. No clear relationship was observed between GC content and validation performance (Pearson r = −0.13, p = 0.6; Spearman ρ = −0.31, p = 0.201).

(**F**) Validation R^2 plotted against the number of training samples. A weak positive trend was observed, but the relationship was not statistically significant (Pearson r = 0.32, p = 0.186; Spearman ρ = 0.39, p = 0.102).
